# Pterygium and Ocular Surface Squamous Neoplasia: Optical Biopsy Using a Novel Autofluorescence Multispectral Imaging Technique

**DOI:** 10.3390/cancers14061591

**Published:** 2022-03-21

**Authors:** Abbas Habibalahi, Alexandra Allende, Jesse Michael, Ayad G. Anwer, Jared Campbell, Saabah B. Mahbub, Chandra Bala, Minas T. Coroneo, Ewa M. Goldys

**Affiliations:** 1 ARC Centre of Excellence for Nanoscale Biophotonics, University of New South Wales, Sydney, NSW 2032, Australia; jam762@uowmail.edu.au (J.M.); a.anwer@unsw.edu.au (A.G.A.); j.campbell@unsw.edu.au (J.C.); s.mahbub@unsw.edu.au (S.B.M.); e.goldys@unsw.edu.au (E.M.G.); 2 Graduate School of Biomedical Engineering, University of New South Wales, Sydney, NSW 2032, Australia; 3 Douglass Hanly Moir Pathology, Macquarie Park, NSW 2113, Australia; aallende@dhm.com.au; 4 Faculty of Medicine and Health Sciences, Macquarie University, Sydney, NSW 2109, Australia; 5 Department of Ophthalmology, Faculty of Medicine and Health Sciences, Macquarie University, Sydney, NSW 2109, Australia; chandrabala.eye@gmail.com; 6 Department of Ophthalmology, University of New South Wales at Prince of Wales Hospital, High Street, Randwick, NSW 2031, Australia; m.coroneo@unsw.edu.au

**Keywords:** pterygium, ocular surface squamous neoplasia, boundary detection, autofluorescence, machine learning

## Abstract

**Simple Summary:**

Cancer is able to damage the surface of the eye, especially in countries like Australia with high exposure to ultraviolet radiation from the sun. Such cancer (ocular surface squamous neoplasia or OSSN) is similar in appearance to a common and benign eye disease called pterygium. Currently, eye biopsy is the gold standard diagnostic method for OSSN, which is traumatic for the patient, carries risks, and has been potentially unnecessary in patients diagnosed with pterygium only. This research introduces an imaging-based method for OSSN screening, which will reduce pressure on health resources—and hopefully eliminate the need for eye biopsy in cases of suspected OSSN.

**Abstract:**

In this study, differentiation of pterygium vs. ocular surface squamous neoplasia based on multispectral autofluorescence imaging technique was investigated. Fifty (N = 50) patients with histopathological diagnosis of pterygium (PTG) and/or ocular surface squamous neoplasia (OSSN) were recruited. Fixed unstained biopsy specimens were imaged by multispectral microscopy. Tissue autofluorescence images were obtained with a custom-built fluorescent microscope with 59 spectral channels, each with specific excitation and emission wavelength ranges, suitable for the most abundant tissue fluorophores such as elastin, flavins, porphyrin, and lipofuscin. Images were analyzed using a new classification framework called fused-classification, designed to minimize interpatient variability, as an established support vector machine learning method. Normal, PTG, and OSSN regions were automatically detected and delineated, with accuracy evaluated against expert assessment by a specialist in OSSN pathology. Signals from spectral channels yielding signals from elastin, flavins, porphyrin, and lipofuscin were significantly different between regions classified as normal, PTG, and OSSN (*p* < 0.01). Differential diagnosis of PTG/OSSN and normal tissue had accuracy, sensitivity, and specificity of 88 ± 6%, 84 ± 10% and 91 ± 6%, respectively. Our automated diagnostic method generated maps of the reasonably well circumscribed normal/PTG and OSSN interface. PTG and OSSN margins identified by our automated analysis were in close agreement with the margins found in the H&E sections. Such a map can be rapidly generated on a real time basis and potentially used for intraoperative assessment.

## 1. Introduction

**Figure 1:**
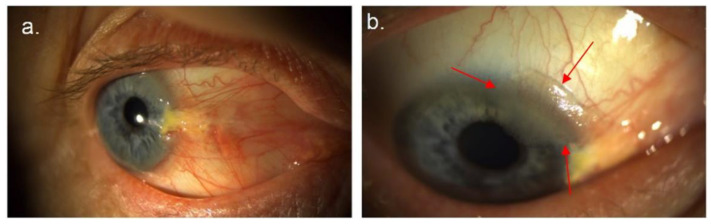
(**a**) Right nasal pterygium with atypical changes at the superior margin. (**b**) Gelatinous lesion (arrows) contiguous with and arising in the super-limbal aspect of the pterygium—confirmed as OSSN by biopsy.

Pterygium (PTG) and ocular surface squamous neoplasia (OSSN) are two potentially coexistent types of ocular surface tumor, which share similar risk factors, most notably exposure to UV light (Figure 1a,b) [1,2], damaging the ocular surface [3,4]. While PTG is a benign, proliferative disorder of the ocular surface, OSSN is a neoplastic disease of the conjunctival/corneal epithelia, ranging from mild dysplasia to invasive squamous cell carcinoma (SCC) [5,6,7]. Ocular surface tumors have a high incidence in regions of high terrain reflectivity [2], including peri-equatorial regions such as Australia where PTG occurs in 7.3% of population [8] and is particularly elevated in older men (appearing in 12% of males over 60 years) [9]. The prevalence of OSSN in pterygia varies between 5–30% and may depend in part on the degree of UV exposure and patient/population immune status [10]. In 9.8% of cases of PTG, histopathological review [11] found the unexpected presence of OSSN. This can cause difficulties in clinical management, as standard treatments for PTG and OSSN are different.

Traditionally, OSSN is routinely removed surgically using a no-touch technique, with 4- to 5-mm margins, with alcohol epitheliectomy to the cornea, and application of cryotherapy to the surgical margins [6]. However, surgery may be associated with OSSN recurrence, and for this reason, topical medical treatment (interferon with or without retinoic acid) is increasingly preferred [12]. The gold standard treatment for PTG, however, is by excision, followed by reconstruction of the excision defect with a free limbal/conjunctival or conjunctival graft [13] with or without intraoperative mitomycin- C [14]. The severe end of the disease spectrum, consequent upon delayed diagnosis and lack of appropriate diagnostics [15,16], may lead to invasive lesions, which are potentially life threatening [17,18] and may require extensive surgery, including exenteration [15].

In current practice, following clinical suspicion, diagnosis relies on confirmation by tissue biopsy [19,20], and histological assessment represents the diagnostic gold standard for PTG and OSSN [19]. A tissue biopsy is invasive and may carry the risk of seeding in malignant cases [21]. An alternative less invasive biopsy method used for OSSN diagnosis is impression cytology, allowing visual assessment of the superficial layers of ocular surface epithelium removed by a cellulose acetate filter [22]. The reliability of impression cytology remains controversial due to the sampling error involved in retrieving cells only from the superficial layers of the ocular surface [23]. As tissue biopsies and impression cytology are only performed in the case of clinical suspicion, small or atypical lesions may be overlooked [24]. Both specimens, histological and cytological, require significant sample preparation time and clinical expertise [25], and are not always reliable for detecting tumor boundaries, hampering adequate margin clearance, which is usually judged by visual inspection at the time of surgery. These techniques will also miss multifocal disease, which may be present in 26% of cases [26]. New imaging modalities for detecting OSSN include in vivo confocal microscopy [27] and anterior segment optical coherence tomography (OCT) [28,29]. Although these sophisticated imaging technologies are non-invasive, they have shown mixed results when their findings are correlated against tissue biopsies [30]. In addition, these technologies have operator dependent drawbacks and limited capability for assessing thick keratotic lesions or superficially invasive carcinomas [30].

Autofluorescence multispectral imaging (AFMI) is a novel and translatable imaging technique developed in our group [31,32,33] whose application to distinguish OSSN from PTG has not previously been investigated. The AFMI technology excites eye tissue using a safe level of light in a number of narrow spectral bands (±5 nm). Tissue autofluorescence in several spectral ranges defined by optical filters is captured, making it possible to obtain autofluorescent spectral signatures of different types of ocular tissue [31]. AFMI is able to distinguish between different types of tissue due to its capacity to recognize aspects of chemical tissue composition based on fluorescence spectral signatures [34,35] of cell-native compounds such as, protoporphyrin IX (PPIX), reduced nicotinamide adenine dinucleotide (NADH) and flavin adenine dinucleotide (FAD) [36,37]. Cancerous cells are, in general, highly glycolytic; producing a large proportion of energy from the fermentation of glucose into lactate regardless of oxygen availability, resulting in changes in the concentration and ratios of fluorophores compared to healthy cells [38,39]. Consequently, the collective changes in concentrations of these fluorophores can be exploited by the AFMI technique to differentiate cells with divergent metabolic states [40]. AFMI has unique advantages for the assessment of ocular surface disease as it is potentially fully non-invasive and contactless, with the added benefit of relative ease of use with little technical training of staff required. AFMI is capable of producing real-time results in an outpatient setting, and has potential for automation, thereby avoiding subjective assessment [31].

In this study, we extend the AFMI methodology [31] to characterize PTG in comparison with normal and neoplastic tissue (OSSN) in fixed human ocular biopsy samples. We utilized an expanded number of channels (*N_ch_* = 59) in comparison to those previously published [31] to facilitate the tracking of key native fluorophores including PPIX, flavins, and lipopigments. We have also introduced a novel paradigm for ocular surface tumor assessment (termed “fused classification framework”), which combines intra- and inter-patient classification [19]. This framework was designed specifically for OSSN/PTG classification allowing it to handle the OSSN/PTG heterogeneity, while minimizing aspects of patients’ variability. Further, objective detection of boundaries between OSSN, PTG, and normal tissues was used to create false color maps, of potential utility for rapid real-time intraoperative assessment. The detection of OSSN/PTG boundaries employing our AFMI-based method were validated by an anatomical pathologist. The application of multispectral analysis of autofluorescence in combination with artificial intelligence, to the best of our knowledge, is a novel approach in identification and boundary determination of human OSSN in the presence of PTG.

## 2. Materials and Methods

### 2.1. Patient Recruitment

**Figure 2:**
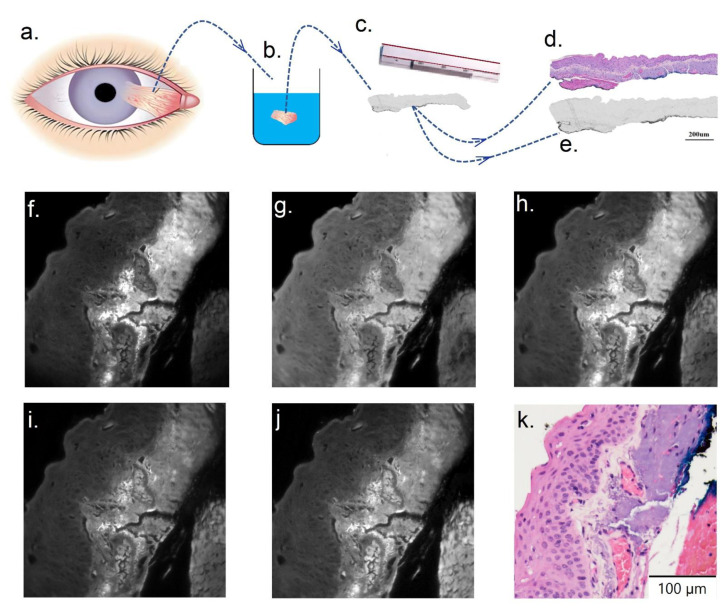
(**a**–**e**) Sample preparation and histological assessment. (**a**) Ocular surface biopsy collected from patients. (**b**) Histology sample processed following formalin fixation into paraffin embedded sections. (**c**) Two adjacent sections were cut using a microtome and then dewaxed. (**d**) Example cut tissue section, which was H&E stained and coverslipped for histology assessment and used as reference. (**e**) The unstained tissue section adjacent to that shown in (**d**). Such sections were placed on a slide, coverslipped, and used for multispectral imaging analysis. (**e**–**j**) Example tissue images in selected channels (channels number 3, 16, 22, 31, and 45, respectively). (**k**) H&E stained section of example tissue shown in (**e**–**j**).

The study was approved by the University of New South Wales Human Research Ethics Committee, project no. HC190310. Patients (N = 50) diagnosed with either OSSN or pterygium undergoing a clinically indicated ocular biopsy were recruited. After collecting ocular surface biopsy samples from patients (Figure 2a), tissue was fixed in formalin, dehydrated, and paraffin embedded (Figure 2b). For each patient, adjacent serial tissue sections were cut and dewaxed (Figure 2c). Serial adjacent sections refer to consecutive slices of tissue that are 4 um in thickness, which are not identical but appear similar as they preserve the x–y spatial axis, but are cut at different z-depths. Adjacent tissue sections were cover-slipped in two groups: with or without hematoxylin and eosin staining (H&E). The stained and unstained tissue are shown in Figure 2d,e, respectively. The unstained section without H&E was used for multispectral imaging and subsequent spectral imaging analysis (Figure 2e). Our analysis was applied only to the epithelial component of the tissue. Based on the reference H&E section brightfield microscopy, a trained pathologist classified specimen images into regions of normal, PTG, and/or OSSN.

### 2.2. Multispectral Imaging

Multispectral microscopy (on an adapted standard fluorescence microscope Olympus iX83™ with a 40× oil U12TM objective) was used with 59 fluorescent channels (details are in Supplementary Table S1). These channels were designed to be able to characterize some of the most abundant endogenous tissue fluorophores [41,42,43,44,45,46,47,48]. A multispectral excitation lamp (from Quantitative™, Sydney, AU) was used with five epifluorescence filter cubes to produce these defined spectral channels (details are in Supplementary Table S1). Images were captured by an electron multiplying charged-coupled device or EMCCD (sensor size was 1024 × 1024 pixels from Nuvu™ 1024, Montreal, Canada) with operating temperature of −60 °C to reduce sensor-induced noise. The image acquisition time was set to an average of 0.8 seconds (averaging the photon count was used if more than one image was captured for a specific channel). The averaging allowed collection of higher quality images (higher signal to noise ratio (SNR) despite using low power LEDs) while minimizing photobleaching of the biological samples. Figure 2f–k illustrate images from example Channels 3, 16, 22, 31, and 45 and the corresponding H&E section of a sample patient, respectively.

### 2.3. Establishing Spectral Signatures 

Spectral images are subjected to image noise such as background fluorescence, illumination curvature, dead or saturated camera pixels, and or Poisson’s noise, which were minimized through image preprocessing [31] (details in Section S1). After image stitching (details in Section S2), to extract quantitative spectral information from multispectral images and generate spectral signatures associated with normal, PTG and OSSN tissue regions, spectral channel images of the tissue were first divided into corresponding sectors (squares, 16 × 16 pixels each). The average intensities of spectral channels were calculated for each sector, which formed an $N_{ch}$ ($N_{ch}=59)$ dimensional vector [49,50]. This 59- dimensional vector is considered to be a spectral signature of the sector. Next, each sector was labelled as normal, PTG or OSSN depending on the pathology review. These spectral signatures were used as data points in subsequent analyses. 

### 2.4. Fused Classification Framework

Generally, two complementary frameworks in the field of image analysis, inter-patient and intra-patient, have been introduced to identify cancer margins [25]. In the intra-patient framework, the training and testing datasets include tissue sections from a single patient, while in the inter-patient framework, the tissue sections for test and training come from different individuals [25]; this is more clinically relevant. Inter patient and intra patient framework have their own advantages and disadvantages. The intra-patient classification framework where the dataset from only a single patient at a time is analyzed, minimizes the interpatient variability, but at the same time, patient heterogeneity is overlooked. Inter-patient classification considers patient heterogeneity at the expense of inter-patient variability with respect to cancer detection.

In this study, we introduced a new framework called fused classification framework, which combines inter- and intra-patient classification [31]. For OSSN/PTG characterization using AFMI, we have a unique opportunity to easily access multispectral image data from a normal section of eye for each patient. As shown in Figure 1a, OSSN and PTG generally occur at a specific position on the eye in the interpalpebral fissure, often at the limbus. Therefore, it is feasible to take an image from a normal area for each patient and to extract the associated normal spectral signature. Having a normal signature from each patient theoretically reduces confounders that may influence the tumor (OSSN or PTG) spectral signature, as per Equation (1).

(1) $$\mathrm{RSS}\;=\frac{SS-Med}{Std}$$

where, RSS, *SS*, *Med,* and *Std* are relative spectral signature, spectral signature, median value of normal spectral signature for a single patient, and standard deviation of spectral signature of normal section. Subsequently, RSS can be used for training machine learning classifiers. We call this framework a “fused framework” as it employs the intra-patient approach in terms of using normalized data points and also inter-patient classification where data from all patients are used to develop classifiers.

### 2.5. Multivariate Analysis and Data Analysis Method

AFMI used in this work employed 59 channels resulting in a high dimensional spectral signature. To uncorrelate and compress the 59-dimensional signature, unsupervised principal component analysis (PCA) was used. PCA is a popular technique for reducing the dimensionality of a dataset, while minimizing information loss via creating new uncorrelated variables that optimally capture data variance [51] (further details in Sections S3 and S4). We transformed the 59 dimensional spectral signature data using PCA and we further considered only five top-ranked PCA scores, which captured >90% of the original data variability [52,53]. Further, these top five PCA scores were used to construct the classifiers. These algorithms automatically categorize compressed spectral signatures into one or more sets of “classes”, e.g., normal, PTG, OSSN, after learning data structure from the training data set. The classifier performance is visualized by a ‘receiver operating characteristic’ (ROC) curve and quantified by the area under ROC curve (AUC) [49]. AUC values close to one indicate excellent performance of the classifier. In this study, support vector machine (SVM) [54,55] was selected, as high performance of this classifier was shown in a recent similar study [56] (further details Section S5). To rigorously validate our analysis and minimize the risk of classifier overfitting, we used two standard cross validation methods [57]: K fold cross validation and the “leave one [patient] out” method [58], which provides unbiased performance assessment using validation testing data points (further details Section S6) [59]. As training, the SVM classifier was based on PCA-reduced set of the first five highest ranked scores, to validate and test the classifier, the unknown input data was reduced to the top five PCA scores before running the SVM classification. 

### 2.6. Statistics

To establish statistical significance of the observed differences, the Mann–Whitney U test (two-tailed test) was applied using Matlab 2018b, while the non-parametric distribution of the data was confirmed [53]. Significant differences were shown with * (for *p*-value < 0.05), ** (for *p*-value < 0.01), and *** (for *p*-value < 0.001).

## 3. Results

### 3.1. OSSN and PTG Classification

**Figure 3:**
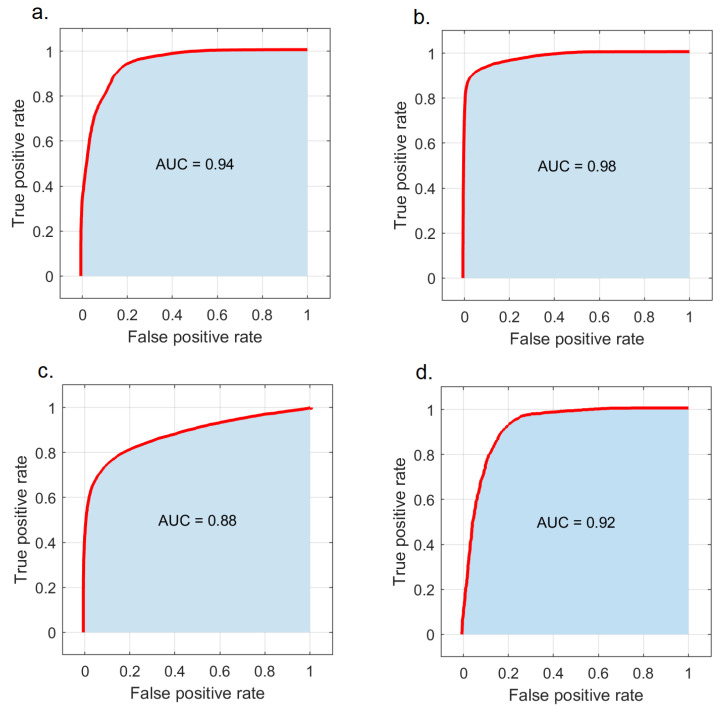
Patient classification performance of the SVM classifier. (**a**) ROC curve obtained from PTG and OSSN classification. (**b**) ROC curve obtained from normalized normal and OSSN classification. (**c**) ROC curve obtained from normal and PTG classification. (**d**) ROC curve obtained from normal, PTG, and OSSN classification.

We analyzed relative spectral signatures (top five PCA variables) between normal, PTG, and OSSN to train the SVM classifier using fused framework classification (see Section 2.4) and construct the corresponding ROC curve shown in Figure 3. The SVM classifier trained to distinguish PTG from OSSN, demonstrated high performance with AUC = 0.94 (Figure 3a). The SVM classifier performance in classifying OSSN vs. normal and PTG vs. normal was found to be AUC = 0.98 and 0.88, respectively (Figure 3b–c). The SVM classifier also showed excellent performance in a three-way classification (normal, PTG, and OSSN) with AUC = 0.92 (Figure 3d). Based on a ten-fold cross-validation approach (Supplementary Material Section S5), the accuracy, sensitivity, and specificity of the classifier to distinguish PTG from OSSN were found to be 88 ± 6%, 84 ± 10%, and 91 ± 6%, respectively. Further, the “leave one patient out” procedure was employed to regressively evaluate this classifier, which showed the overall accuracy was 81%.

### 3.2. OSSN and PTG Spectral Signature Visualization

**Figure 4:**
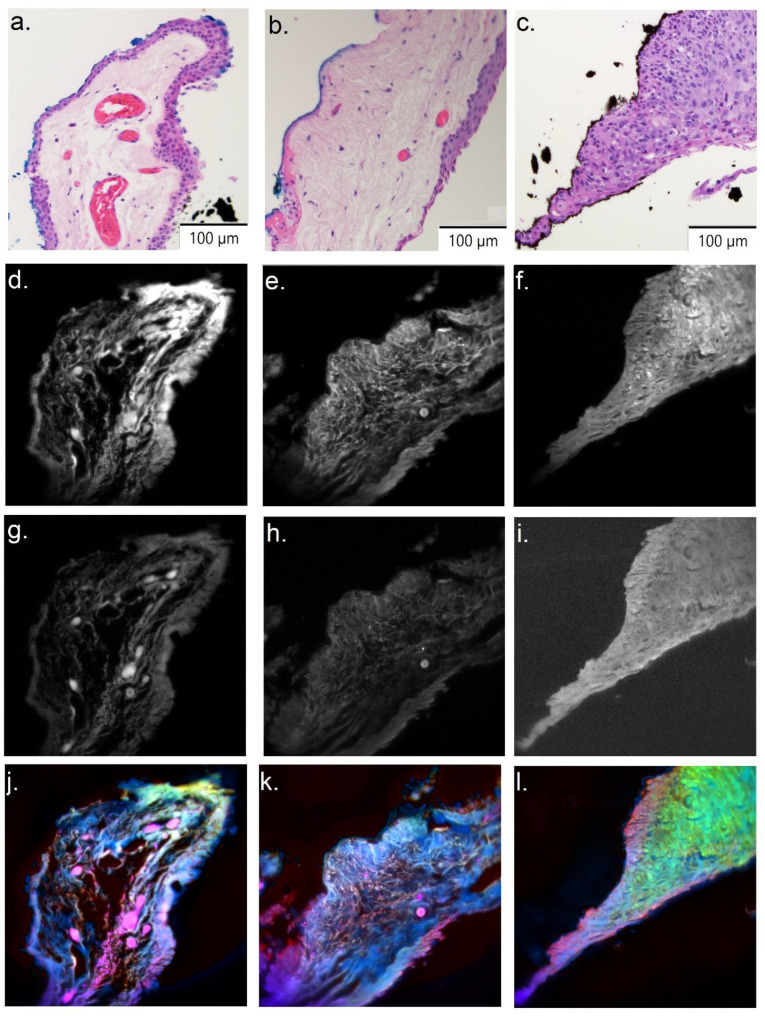
Spectral differences between normal, PTG, and OSSN. (**a**–**c**) H&E image for normal, PTG, and OSSN sections, respectively. (**d**–**f**/**g**–**i**) Channel 1/Channel 20 for normal, PTG, and OSSN sections, respectively. (**j**–**l**) PCA false color image for normal, PTG, and OSSN sections respectively. OSSN is green, while normal and PTG are violet.

Figure 4 shows normal, PTG, and OSSN sections from a sample patient. Figure 4a–c shows the H&E images references for normal, PTG, and OSSN, respectively. Figure 4d–f and g–i represent a single channel image (Channel 1/Channel 20), which shows intensity variations in normal, PTG, and OSSN, respectively. Spectral distinctions between tissue signatures are visible in the false color images of normal (Figure 4j), PTG (Figure 4k), and OSSN sections (Figure 4l). In the PCA projection used here (details in Supplementary Material Section S4), the normal, PTG, and OSSN false color section are visualized by yellow, blue, and green color, respectively. Pure pink colors represent red blood cells within vessels, which point to the consistency of our analysis.

### 3.3. Fluorophore Analysis

AFMI employs tens of channels with excitation/emission wavelengths matching the excitation/emission patterns of common fluorophores in the tissue. There are several highly abundant tissue fluorophores, and it is possible for some channels to be dominated by a particular fluorophore [34,35]. 

**Figure 5:**
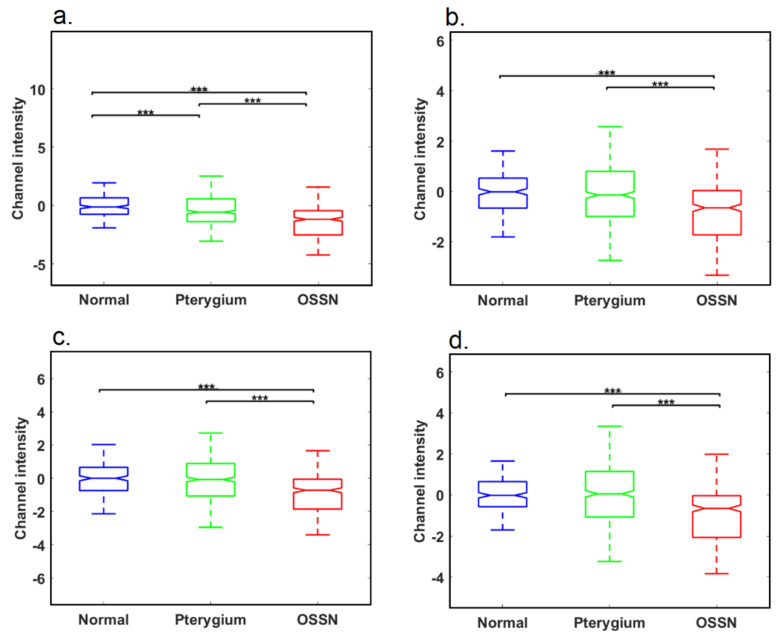
Analysis of fluorophore signals in the tissue. (**a**) Intensity analysis for Channel 3 containing a contribution from elastin. (**b**) Intensity analysis for Channel 12 containing a contribution from lipopigment. (**c**) Intensity analysis for Channel 30 containing a contribution from flavins. (**d**) Intensity analysis for Channel 52 tentatively attributed to PPIX (*** represents *p*−value < 0.01). Corresponding sample channels are shown in Supplementary Figure S5.

Figure 5 shows relative intensity values of channels 3, 12, 30, 52 (calculated using Equation (1)), in PTG, OSSN, and normal tissue regions delineated as above. These values are tentatively attributed to contributions from elastin, lipopigment, flavins, and PPIX (further details in Section S7 and Supplementary Figures S2–S4). We found that the spectral signal in Channel 3 (attributed to elastin fluorescence) was significantly different between normal, PTG, and OSSN tissue (Figure 5a), with reductions demonstrated between normal and PTG, normal and OSSN, and PTG and OSSN. The signal in Channel 12 (attributed to the lipopigment) was reduced between normal and OSSN, as well as between PTG and OSSN, but the difference in the lipopigment signal between normal and PTG was not significant (Figure 5b). An identical pattern was seen for Channels 30 and 52 containing contributions from flavins and PPIX (Figure 5c,d). 

### 3.4. Boundary Detection 

**Figure 6:**
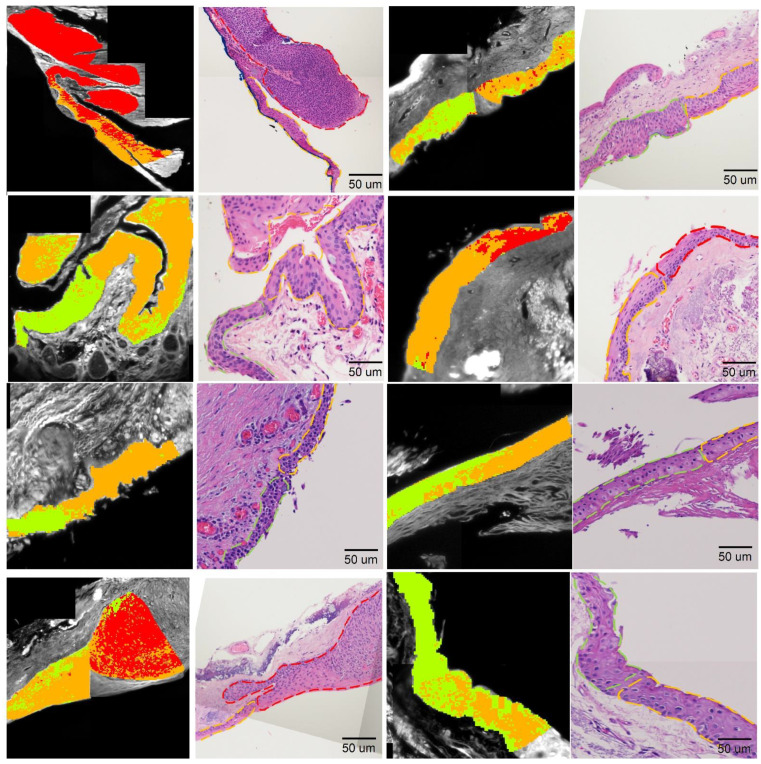
False color map generated to locate the normal, PTG, and OSSN boundary on the testing section based on intra-patient classification framework compared to the associated histology images. The block data position on a single spectral channel (Chno = 10) image is colored in red/orange/green if they are predicted to be OSSN/PTG/normal. First/third column is the multispectral false-color map. Second/forth column is corresponding H&E section with red, orange, and green dash lines highlighting OSSN, PTG, and normal section.

To draw the boundaries of normal, PTG, and OSSN tissues, we evaluated relative differences between neoplastic, PTG, and normal tissue using the intra patient classification framework (flowchart of the method and further details in Section S8). In this framework, the multispectral signature of OSSN, PTG, and normal tissue was established (for each single patient at a time) and used to train the SVM classifier, and then the classifier was applied to region intersections to define OSSN and PTG or normal borders. To create the false color map highlighting the OSSN, PTG, and normal areas, firstly, the boundary sectors were obtained and fed into the classifier procedure to label prediction: normal, OSSN, or PTG. Then, sector positions were colored red (OSSN), orange (PTG), or green (normal) on a composite image obtained from channel No10 (Figure 6, first and third column). 

Further, we evaluated our false-color map against H&E sections (Figure 6 second and fourth column), where H&E images were segmented with dash lines based on pathologist recommendation (Green dash line: normal section, red dash line: OSSN and orange dash line: PTG). Figure 6 shows a close correlation between the multispectral estimation and histopathological assessment at the interface for all of the eight intersections used in this approach. The agreement between our multispectral analysis and H&E can be enhanced by a supervised adjustment to reclassify areas that are obviously misclassified such as removing slight misclassified spots and smoothing the color map. 

## 4. Discussion

PTG is a prevalent ocular surface disease traditionally described as a benign growth of the altered conjunctiva invading the cornea. Recently considered to be induced by an aberrant wound healing process, PTG is characterized by centripetal growth of a leading edge of altered limbal epithelial cells, followed by a squamous metaplastic epithelium with goblet cell hyperplasia and an underlying stroma of activated, proliferating fibroblasts, neovascularization, inflammatory cells, and extracellular matrix remodeling [60]. It can impair sight via a number of mechanisms, is associated with dry eye syndrome and impacts cosmesis [13]. In addition, in approximately 10% of cases PTG coexists with OSSN [5], a neoplastic ocular surface disease with potentially more serious implications [11]. The clinical symptoms and appearance of OSSN and PTG often have similarities [61], which makes definitive diagnosis challenging at times [15,16] and which may result in inappropriate or delayed treatment. Currently, neither clinical examination nor in vivo confocal microscopy can reliably distinguish OSSN from benign conjunctival pathology [9] and high-resolution OCT is not widely available, potentially resulting in missed diagnoses of ocular surface malignancy.

Our group recently introduced an imaging method (AFMI) that uses detailed spectral images at defined multiple excitation and emission wavelength ranges, to distinguish normal tissue from OSSN, which has a specific spectral signature. However, differentiation of OSSN and PTG was not investigated. In this study, we extended our previous work to characterize normal, OSSN, and PTG by AFMI. We advanced our previous AFMI system by adding more spectral channels (*N_ch_* = 59) to extract detailed spectral information from our samples. This made it possible to evaluate the overall biochemical composition in the tissue sections more accurately and also to help in identifying single biomarkers capable of classifying PTG and OSSN more precisely compared to our previous work [62]. To analyze the spectral signatures of the data, machine learning classifiers were employed, to automatically provide rapid results of AFMI, and avoid the requirement for highly skilled operators for image interpretation. The outcomes of the AI were evaluated against pathological assessment and showed reliable results, which were in close agreement with expert evaluation. 

We carried out the comparison of autofluorescence images from spectral channels most closely associated with the signals of specific endogenous tissue fluorophores: extracellular matrix protein elastin (Channel 3), lipopigment (Channel 12), flavins (Channel 30), and PPIX (Channel 52). The results suggest that signals in Channel 3 were statistically different between normal and PTG tissue (Figure 1a). Prior findings have indicated dysregulation of elastin in the ECM of (sub-epithelial) PTG tissue [63], which is what we detected by assessment of autofluorescence. As neoplasia results in degradation of the ECM by matrix metalloproteinases [64,65], the more exaggerated decrease of elastin in OSSN tissue would also be unsurprising. Our finding of a decrease of signal in Channel 3 (tentatively attributed to flavins) in OSSN compared to normal and PTG is reinforced by prior research, which showed autofluorescence loss related to a reduced flavins’ signal [66]. However, our (tentative) observation that PPIX, was depleted in OSSN has not been previously reported and will require further investigation. PPIX can be elevated in some neoplastic tissue, to the point that it is used as a cancer biomarker and for the definition of tumor boundaries [67]. Of interest is that increased levels of transketolase-like-1-gene (TKTL1) protein have been reported in OSSN [68], an indication of enhanced glycometabolism in these lesions. Furthermore, enhanced TKTL1 expression seemed to predict clinical outcomes, especially the tumor recurrence rate. This is consistent with previous work on enhanced glycolysis and altered fluorophore levels [34,35]. While glycolysis in pterygium does not appear to have been investigated our findings could suggest lower levels of activity, hence our ability to detect spectral differences. Lipopigment has been found to be elevated with age as well as in other ocular diseases [69,70]. The increased signal from Channel 12 indicated that there was an elevation of lipopigment in OSSN tissue relative to normal, but this was not reflected in PTG. This effect cannot be attributable to differences in age as normal and tumor tissues were drawn from the same donors. 

In this study, we used a novel classification framework called fused-framework to minimize the effects of biological heterogeneity of patients [71]. This framework is based on normalizing each patient data to the image of their own normal tissue and showed higher classification performance (AUC = 0.94) compared to conventional interpatient classification with AUC found to be 0.79 (Section S9, Supplementary Figure S6). Our analysis was conducted only on the epithelial region of the tissue. Therefore, although we investigated fixed tissue sections from biopsies, our approach is feasible and would be practical in a clinical setting, where an ophthalmologist would be able to image normal epithelial areas either from the unaffected contralateral eye or from the diseased eye, including eyes with evidence of multifocal disease virtually in real time [72].

Rapid automated identification of boundaries of OSSN and PTG is important for treatment monitoring. During surgical excision, accurate boundary detection can help a surgeon to completely excise the tumor, which reduces the risk of subsequent recurrence and/or tumor-related morbidity [73]. In the past, patients experienced a high recurrence rate after surgery due to incomplete excision [74,75], although newer medical treatment regimens are associated with a higher success rate [12,76]. In addition, precise boundary recognition would assist the surgeon to preserve as much healthy tissue as possible, preventing redundant resection. If topical treatment is used for OSSN, early treatment termination due to a false clinical impression may increase recurrence risk, which our boundary detection approach may help to avoid.

## 5. Conclusions

AFMI presents unique benefits in ophthalmological applications. Clinical translation of the technology is possible due to the simple instrumentation, and contactless nature, making AFMI compatible with diagnostics in an outpatient setting. AFMI technology can be simply combined with a slit lamp in the clinic for assessment or be integrated with surgical equipment if surgical intervention is necessary. We also emphasize that in this work we employed safe low excitation powers posing no harm to patients (Supplementary Table S1). AFMI is relatively inexpensive, which is an advantage over ultrasound biomicroscopy and confocal microscopy [24] and the technology is time efficient and user friendly with limited need for technical expertise, which would prove invaluable in guiding clinical management. AFMI can also provide insights into the underlying molecular pathophysiology of PTG and OSSN by quantification of metabolic molecular components such as NADH and FAD, and also perhaps lend itself to evaluating limbal epithelial crypt metabolism in investigating the potential role of the progenitor cells in PTG and OSSN pathogenesis [77] to help us better understand the disease mechanism in OSSN. Limitations of our study include the use of paraffin-embedded ex vivo tissue, so that the results presented here are valid only in this scenario. Another limitation is the tentative assignment of fluorophores to key channels, which have yet to be confirmed by direct tissue staining. 

## Data Availability

Data are available upon reasonable request.

## Supplementary Materials

The following supporting information can be downloaded at: https://www.mdpi.com/article/10.3390/cancers14061591/s1, Figure S1: Image registration for a sample patient spectral image (Ch10) according to corre-sponding bright field image and epithelium segmentation for subsequent analysis; Figure S2: (a) Elastin excitation and emission spectra (blue and red line) and excitation emission wavelength range of channel 3 (yellow and green band). (b) Lipopigment excitation and emission spectra (blue and red line) and excitation emission wavelength range of channel 12 (yellow and green band). (c) Flavins excitation and emission spectra (blue and red line) and excitation emission wavelength range of channel 30 (yellow and green band). (d) PPIX excitation and emission spectra (blue and red line) and excitation emission wavelength range of channel 52 (yellow and green band); Figure S3. Spectral images associated to elastin, lipofuscin, flavins and PPIX. First column/ second column/ third column is normal/ PTG/ OSSN section. Corresponding H&E section for normal, PTG and OSSN are shown in Figure 4a–c, respectively. (a–c) Elastin channel. (d–f)Lipopigment channel. (g–i) Flavins channel. (j–l) PPIX channel; Figure S4. Classifier perfor-mance for differential diagnosis of Normal, PTG and OSSN using elastin, lipopigment, flavins and PPIX corresponding channels; Figure S5: Intra-patient framework flowchart to determine the OSSN/PTG/normal; Figure S6: Inter patient classification performance (AUC = 0.79); Table S1: Details of spectral channels used in multispectral imaging; Section S1: Image Stitching; Section S2: Principal Component Analysis (PCA); Section S3: Multispectral Image Visualization; Section S4: Support Vector Machine Classifier; Section S5: Cross Validation; Section S6: Single Channels At-tribution to Fluorophores; Section S7: Intra Patient Classification; Section S8: Inter Patient Classi-fication; Section S9: Inter Patient Classification. References [31,32,34,35,40,43,44,45,49,51,56,58,62,78,79,80,81,82,83,84,85,86] are referred to in Supplementary Materials.
