# Supplementary material for: Pterygium and Ocular Surface Squamous Neoplasia: Optical Biopsy Using a Novel Autofluorescence Multispectral Imaging Technique
Source: Cancers (Basel). 2022 Mar 21;14(6):1591. doi: 10.3390/cancers14061591 (PMC8946656; doi:10.3390/cancers14061591)
Supplement: Supplementary file 1 [file cancers-14-01591-s001.zip › cancers-1581002-supplementary.pdf]

SUPPLEMENTARY MATERIAL

Table S1. Details of spectral channels used in multispectral imaging.

Channel Number	Excitation wavelength(±5) [nm]	Emission wavelength [nm]	Averaging	Exposure time (s)	Power (μw)
1	'345'	'414'	'4'	'0.4'	1.20
2	'345'	'451'	'4'	'0.4'	1.23
3	'345'	'575'	'2'	'1.0'	1.17
4	'490'	'575'	'1'	'2'	5.24
5	'505'	'575'	'3'	'1.0'	11.81
6	'345'	'594'	'2'	'1.5'	1.26
7	'490'	'594'	'1'	'2'	5.39
8	'505'	'594'	'2'	'1.0'	12.16
9	'345'	'675'	'1'	'3.5'	0.95
10	'490'	'675'	'1'	'3.5'	4.52
11	'505'	'675'	'1'	'2.5'	10.22
12	'358'	'414'	'4'	'0.4'	1.86
13	'371'	'414'	'4'	'0.4'	0.72
14	'377'	'414'	'4'	'0.4'	0.41
15	'358'	'451'	'4'	'0.3'	1.85
16	'371'	'451'	'4'	'0.4'	0.71
17	'377'	'451'	'4'	'0.4'	0.42
18	'371'	'575'	'3'	'1.0'	0.95
19	'377'	'575'	'4'	'0.4'	1.16
20	'381'	'575'	'4'	'0.4'	2.77
21	'385'	'575'	'4'	'0.4'	3.12
22	'391'	'575'	'4'	'0.4'	2.36
23	'400'	'575'	'4'	'0.4'	3.68
24	'403'	'575'	'4'	'0.4'	4.63
25	'406'	'575'	'4'	'0.4'	3.41
26	'412'	'575'	'4'	'0.4'	4.66
27	'418'	'575'	'4'	'0.4'	3.95
28	'430'	'575'	'4'	'0.4'	4.00
29	'437'	'575'	'4'	'0.4'	5.15
30	'451'	'575'	'4'	'0.4'	6.47
31	'457'	'575'	'4'	'0.4'	5.04
32	'469'	'575'	'3'	'0.5'	4.30
33	'476'	'575'	'3'	'0.5'	5.21
34	'371'	'594'	'2'	'1.0'	1.00
35	'377'	'594'	'1'	'3.5'	1.22
36	'381'	'594'	'1'	'2.0'	2.92
37	'400'	'594'	'1'	'2.0'	3.85
38	'403'	'594'	'1'	'2.0'	4.84
39	'406'	'594'	'1'	'2.0'	3.57
40	'412'	'594'	'1'	'2.0'	4.87
41	'418'	'594'	'1'	'2.0'	4.12
42	'430'	'594'	'1'	'2.0'	4.18
43	'437'	'594'	'1'	'2.0'	5.38
44	'457'	'594'	'1'	'2.0'	5.25
45	'469'	'594'	'2'	'1.0'	4.46
46	'476'	'594'	'2'	'1.0'	5.41
47	'371'	'675'	'1'	'2.5'	0.82
48	'377'	'675'	'2'	'1.5'	0.99
49	'381'	'675'	'2'	'1.0'	2.39

50	'400'	'675'	'2'	'1.0'	3.17
51	'403'	'675'	'2'	'1.0'	3.96
52	'406'	'675'	'2'	'1.5'	2.91
53	'412'	'675'	'3'	'1.5'	3.99
54	'418'	'675'	'3'	'1.0'	3.36
55	'430'	'675'	'2'	'1.5'	3.43
56	'437'	'675'	'2'	'1.0'	4.46
57	'457'	'675'	'2'	'1.5'	4.36
58	'469'	'675'	'1'	'2'	3.71
59	'476'	'675'	'1'	'2'	4.49
60	Bright field image				

Section S1: Image Stitching

In this work we used a microscope objective at 40 times magnification with a limited field of view (~ 0.20 -0.25 mm2 of a sample is captured in each image compared with sample area of ~ 5 mm2). To enable the evaluation of a large field of view, we took a series of overlapping spectral images. then, the overlapping images were co-registered using the affine algorithm as per Reference [31] to match an image captured by 10x objective with a bright field microscope. Finally, a composite image was produced as shown in Supplementary Figure S1. The spectral signatures analysed in this work were limited only to the epithelial tissue delineated with purple lines on spectral channel 10 image in Supplementary Figure S1.

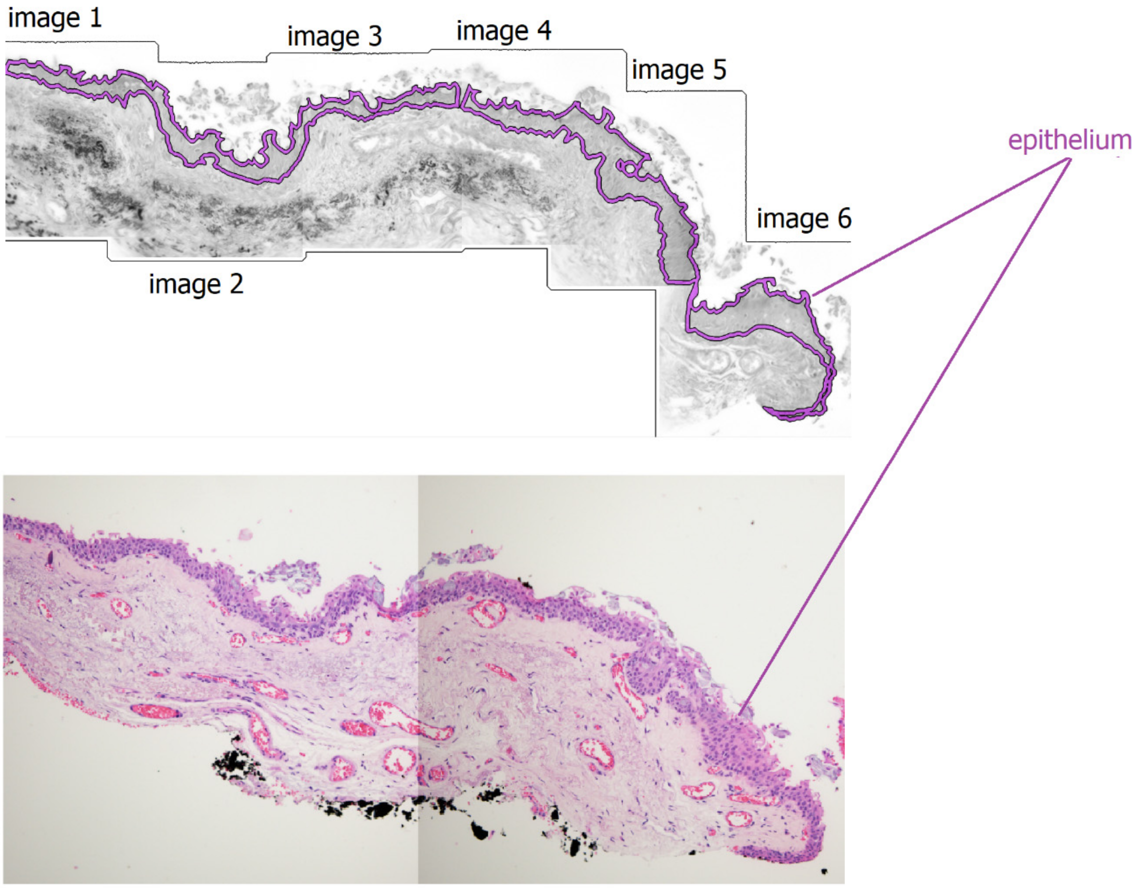

Figure S1. Image registration for a sample patient spectral image (Ch10) according to corresponding bright field image and epithelium segmentation for subsequent analysis.

Section S2: Principal Component Analysis (PCA)

PCA is a common dimensionality reduction methodology which reflects the feature vectors (here elements of spectral signature) onto the eigenvectors of the covariance matrix to illustrate informative variations of uncorrelated data [51]. Using PCA analysis, we reduced the spectral signature dimension from 59 to 5 using the 5 top ranked PCA scores which captured over 90% of data variance.

Section S3. Multispectral Image Visualization

To visualize the variations of spectral tissue signatures across all spectral channels (N=59), the PCA procedure was employed again, to compress all channels to a false color RGB image. The three top-ranked PCA variables created by the PCA transform of autofluorescence intensities from 59 spectral channels in each pixel were colored red, green and blue to create a RGB image

Section S4: Support Vector Machine Classifier

A support vector machine classifier (SVM) was used in this study. This robust supervised technique can effectively deal with sparse data with a minimised risk of overfitting. In this method, a hyperplane was formed [27] with maximum edges in the high dimensional spectral feature space to separate data points into the groups under consideration (here, OSSN, PTG and normal). Then the classifier was trained using the top 5 PCA scores to predict the pre-defined data labels (here, OSSN, PTG and normal).

Section S5. Cross Validation

Cross validation undertaken in this study was based on two standard methods: K fold cross validation and "leave one [patient] out" method [58]. To protect the classifier from the risk of overfitting, K fold (K=10) cross-validation was employed. To this end, the training data set was randomly divided into 10 roughly equal sized subsets (folds) [78]. The classifier procedure was trained by 9 of these folds and evaluated by the one remaining fold. The process was repeated for 10 iterations, and the average accuracies were calculated to obtain the overall accuracy [56]. To apply the "leave one [patient] out" approach [79], a single patient (named the "testing" patient) was selected and their data were put aside. Further, the multispectral signatures of the Normal, PTG and OSSN tissue were established using the remaining cohort of patient data (named "training" patients). This ensures the tissue from the "testing" patient has no impact on the PTG/OSSN signature identified in this step. Furthermore, the established OSSN/PTG signature was used to predict block labels of the "testing" patient. This process was then repeated until all patients considered as testing patients had been treated.

Section S6: Single Channels Attribution to Fluorophores

There are a number of endogenous physiological fluorophores in cells and tissues including (but not limited to) nicotinamide adenine dinucleotide (NADH), NADH phosphate (NADPH), retinoids including N-retinylidene-N-retinylethanolamine (A2E), flavin adenine dinucleotide (FAD) and flavin mononucleotide (FMD, cytochrome C, collagen and elastin. Fluorophores are fairly uniquely characterized by their specific pattern of the excitation/emission spectra [80]. On the other hand, native fluorophores in cells are co-located, at every pixel in the image and their excitation/emission spectra may have a degree of overlap, which makes it difficult to identify specific fluorophores from the relatively featureless net autofluorescence spectrum. If two or more fluorophores have overlapping excitation/emission spectra, it may be impossible to find a channel where only one of them is represented, due to persistent contribution (bleedthrough) from the other(s).

AFMI employs tens of narrow band excitation emission wavelength channels which facilitates to identify the channels dominated by a particular fluorophore [34,35]. To identify channels with minimized effect of signal bleedthrough, we identified common tissue fluorophores with limited spectral overlaps such as PPIX and elastin based on

published excitation emission spectra shown in Figure 1 of Reference [40]. In addition, as highly abundant tissue fluorophores, flavin and lipopigment tend to yield strong signals, specific channels with excitation/emission wavelengths coinciding with these fluorophore excitation/emission patterns were selected. We hypothesise that these channels contain a significant proportion of signals from these fluorophores in the tissue[35], however we are unable to rule out the contributions from other residual fluorophores in combination..

We characterised these four abundant tissue fluorophores (elastin, lipofuscin, flavins and PPIX) showing a strong signal in tissue spectral signature ($p<0.001$) based on their excitation / emission spectra shown in Supplementary Figure S3 (a-d), respectively. Supplementary Figure S3 shows that excitation/emission maxima of elastin/ lipopigment/ flavins/ PPIX are at 355/ 575/ 560/ 675 nm (second peak) and 425/ 575/ 560/ 675 nm, respectively. We hypothesised hat signals in channels 3, 12, 30, 52 have significant contributions from elastin, lipofuscin, flavins and PPIX, respectively, as excitation / emission specifications of these channels (yellow/ green bands in Supplementary Figure S3) match with theses fluorophore excitation/emission maxima (blue/ red lines in Supplementary Figure S3) with limited bleed-through to these channels from other fluorophores. However, we acknowledge that there might be some proportion of signal bleed through in some these channels from other fluorophores. For example, Channel 3 attributed to elastin in this study has some contribution from the flavin signal although the evaluation of the excitation emission spectra of these fluorophores in published literature [40], indicates that a significant portion of the Channel 3 signal is related to elastin.

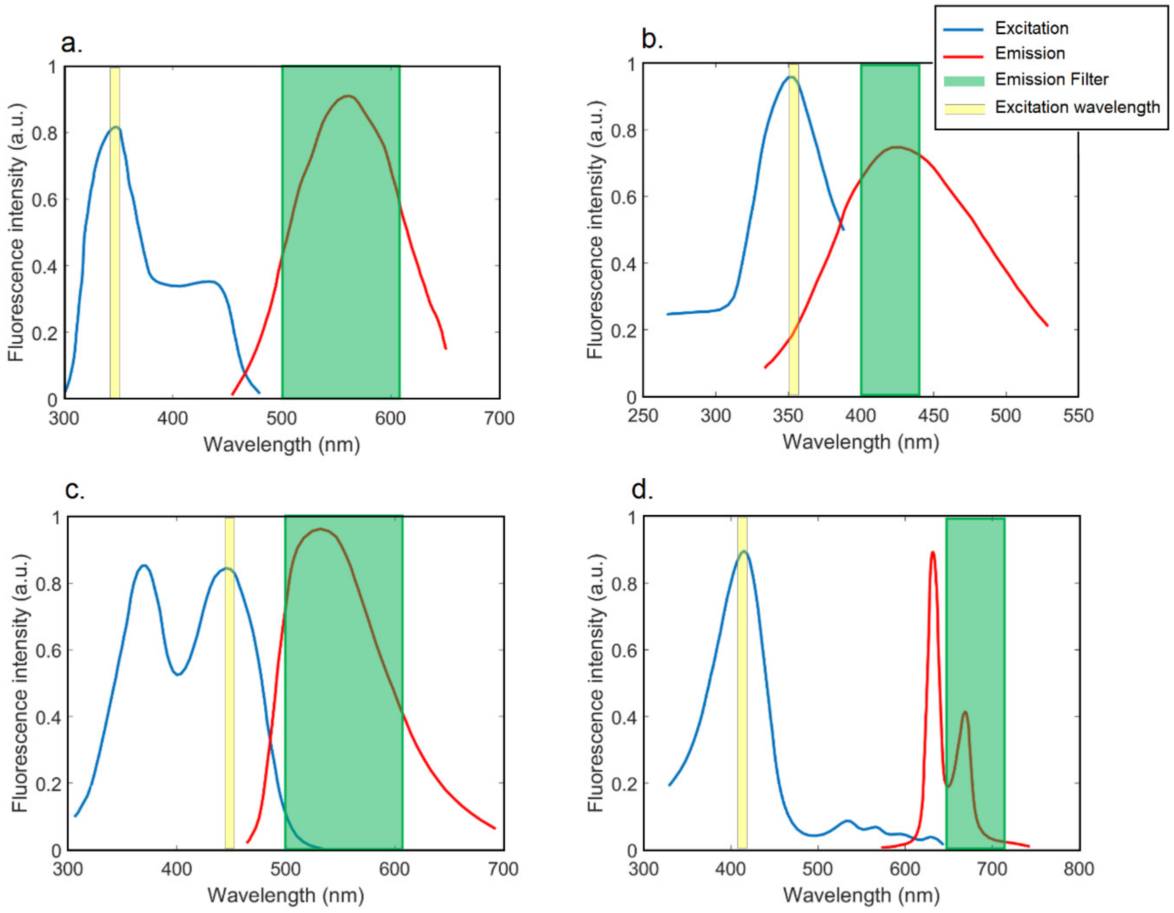

Figure S2. (**a**) Elastin excitation and emission spectra (blue and red line) and excitation emission wavelength range of channel 3 (yellow and green band). (**b**)Lipopigment excitation and emission spectra (blue and red line) and excitation emission wavelength range of channel 12 (yellow and green band). (**c**) Flavins excitation and emission spectra (blue and red line) and excitation emission wavelength range of channel 30 (yellow and green band). (**d**) PPIX

excitation and emission spectra (blue and red line) and excitation emission wavelength range of channel 52 (yellow and green band) [40, 81].

Section S7: Intra Patient Classification

In this study, tumor boundaries were identified based on intra-patient framework. In this framework, the multispectral signature of OSSN/PTG/normal tissue was recognized based on a single patient independently of other patients and then tested on another part of tissue from the same patient which included an intersection of OSSN/PTG or normal. As the training and testing data set was obtained from a single patient, interpatient variability is minimized [25]. To execute this approach, the tissue obtained from a single patient is first split into two separate sections called the training and testing tissue. Depending on the testing intersection tissue pathology, the training data set has data points from both sides. After training the classifier, it was applied to the data blocks of the testing area of the same patient for the label prediction (Normal, OSSN, PTG). Depending on the classifier prediction, the position of the data block was coloured in green, red and orange corresponding to normal, OSSN and PTG label, respectively, thus creating a false colour map. This process was repeated for all patients (n=8) whose samples had an intersection such as PTG / OSSN and enough amount of training data points to properly recognize the associated multispectral signatures.

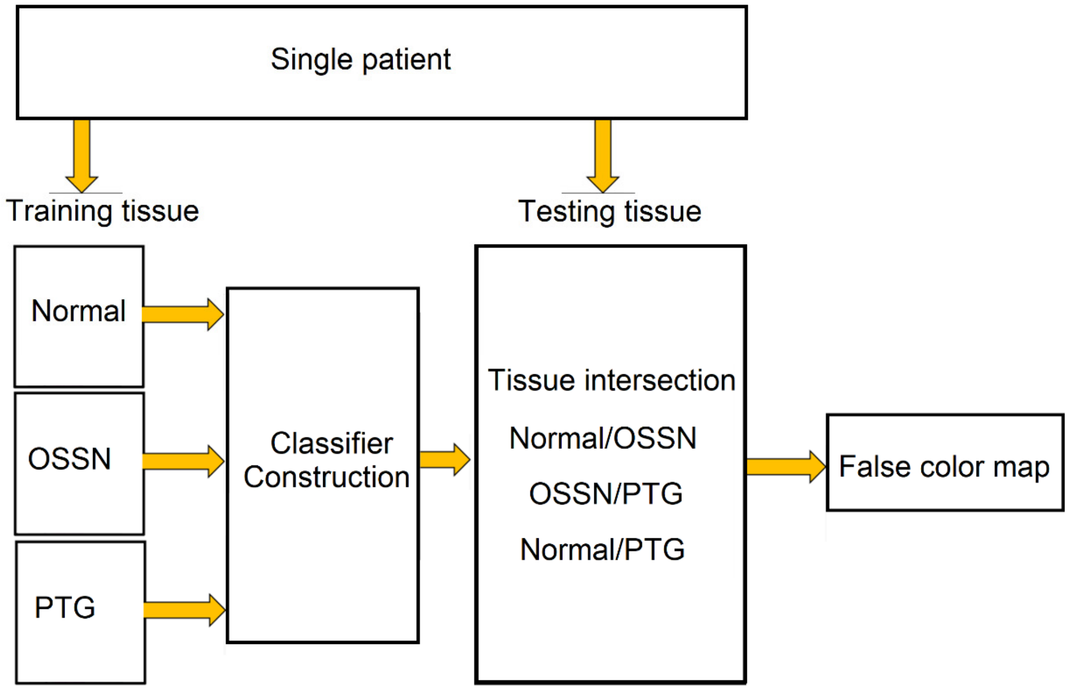

Figure S3. Intra-patient framework flowchart to determine the OSSN/PTG/normal. A single patient tissue sample is first separated into two specific sections named testing and training tissue. The testing tissue contains boundaries between normal/ OSSN, OSSN/PTG or Normal/PTG. The training tissue has areas of tissues including normal, OSSN or PTG and areas, which enables extracting the multispectral neoplastic / PTG signature. Next, data blocks of the testing area were fed to the trained classifiers to predict the labels (Normal, OSSN, PTG) which resulted in a false color map.

Section S8: Inter Patient Classification

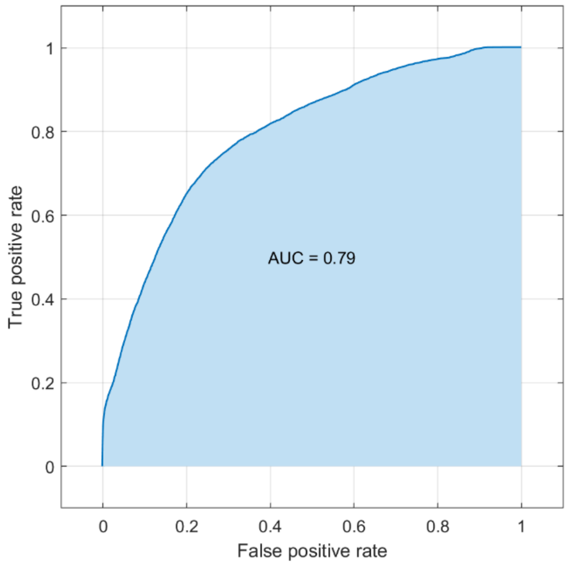

Figure S4. Inter patient classification performance (AUC=0.79).
